# Estimation of genotype by temperature-humidity index interactions on milk production and udder health traits in Montbeliarde cows

**DOI:** 10.1186/s12711-023-00779-1

**Published:** 2023-01-19

**Authors:** Aurélie Vinet, Sophie Mattalia, Roxane Vallée, Christine Bertrand, Beatriz C. D. Cuyabano, Didier Boichard

**Affiliations:** 1 Université Paris Saclay, INRAE, AgroParisTech, GABI, 78350 Jouy-en-Josas, France; 2 Institut de l’Elevage, 75012 Paris, France; 3 INRAE, US310 CTIG, Domaine de Vilvert, 78350 Jouy-en-Josas, France

## Abstract

**Background:**

Heat stress negatively influences cattle welfare, health and productivity. To cope with the forecasted increases in temperature and heat waves frequency, identifying high-producing animals that are tolerant to heat is of capital importance to maintain milk production. This study, based on the joint analysis of on-farm performance and weather data, had two objectives: (1) to determine the response in production performances (milk, fat and protein yields, fat and protein contents) and udder health (somatic cell score) to temperature-humidity index (THI) variations in Montbeliarde cows, and (2) to estimate the interactions between genotype and THI, to enable the identification of the most adapted animals for facing the expected increases in temperature.

**Results:**

Test-day records from first and second lactations from 2016 to 2020 were associated with the average THI during the three days before the test-day record. In total, 446,717 test-day records from 55,650 cows in first lactation and 457,516 test-day records from 58,229 cows in second lactation were analysed. The optimal THI was below 55 (i.e. ~ 12–13 °C) for all traits. Individual responses to THI were estimated by random regression models, which also included individual responses to days in milk. Regardless of the stage of lactation, genetic correlations along the THI gradient were above 0.80, which suggests that genotype-by-THI interactions were weak for production and udder health traits. Nevertheless, a variability in the individual slope of decay could be highlighted at high THI. The genetic correlation between production level at moderate THI and the slope at high THI was negative, while for somatic cell score, it was positive, indicating that heat stress amplifies the susceptibility to mastitis.

**Conclusions:**

The optimal THI for French Montbeliarde cows is below 55 for production and udder health traits. Genetic-by-THI interactions are weak in French Montbeliarde cows for production and udder health traits, but not all animals react in the same way to high temperatures. Even if there is little room for improvement, using a heat tolerance index in cattle selection would be relevant to anticipate the expected increases in temperature. Further investigations are needed to interpret this variability on production traits. However, the current selection for mastitis resistance seems appropriate to adapt cattle to rising temperatures.

**Supplementary Information:**

The online version contains supplementary material available at 10.1186/s12711-023-00779-1.

## Background

Global warming has and will have major negative consequences for livestock production, in particular the increase in the frequency and intensity of heat waves [1]. Indeed, even if breeds remain in the regions where they are currently raised, they will have to face this increase in temperatures. Lactating dairy cows generate a large metabolic internal heat load due to ruminal fermentations and high milk production, and this is exacerbated when the temperature of the environment to which the animals are exposed increases. When body temperature rises, feed intake declines, and consequently, so does the productivity, welfare and health of cows [2, 3].

The Montbeliarde breed is raised throughout France, with a stronger presence in the mid-mountain regions of eastern and central France [4], where average temperatures are below 0 °C from December to February and around 20 °C in July [5]. However, studies on the adaptation of this breed in warmer countries such as Algeria [6], Morocco [7], or Mexico [8] have shown that Montbeliarde cows can acclimatize rather well to higher external temperatures. For instance, the production levels of purebred cows reared in open stalls in Morocco are close to those observed in France and their productive life is longer [7]. However, information on the genetic determinism of heat tolerance, especially in the Montbeliarde breed, is lacking. Thus, to cope with the on-going climate changes, identifying high-producing animals that are also tolerant to heat stress is of capital importance to maintain sustainable milk production.

To perform selection for heat tolerance, indicators of heat stress are needed. There are two main approaches to quantify heat stress. The first one consists in measuring the body temperature or other physiological parameters, such as hormonal response to heat, or measuring behavioral indicators, such as the respiration rate, panting or sweating, which are commonly observed in heat stress events (see [9–11] and reviews in [2, 3]). While these measures provide precise information about the onset of heat stress, they are difficult and often costly to measure at large-scale farming levels, and their deployment as phenotypes to create a reference population with tens of thousands of animals for genomic selection is challenging, even with the currently developing sensor technologies. The second approach to quantify heat stress is based on indirect measures, through analyses of dairy production or reproductive performances of animals. Cattle display maximum genetic potential for production with minimal physiological costs only within their thermoneutral zone (see review in [12]). When the ambient temperature exceeds this thermoneutral zone, the animals spend energy to maintain a normal body temperature, which induces heat stress, and thus alters their performances [12]. Therefore, the association of the decline in performances with the corresponding meteorological conditions informs on the onset of heat stress. Various meteorological indexes based on different combinations of several weather variables (temperatures, humidity, solar radiation or wind speed) have been evaluated as potential predictors of heat stress [13, 14]. The temperature-humidity index (THI) proposed by the National Research Council [15] is highly correlated with the other indexes tested [14, 16] and is now widely used in bovine research, which facilitates the direct comparison of literature data regarding heat stress occurrence with performance losses. THI depends primarily on temperature, but because a high level of humidity emphasizes high and low temperature effects, THI is modulated by relative humidity as a secondary factor.

This study had two objectives: (1) to identify the values of THI associated with the onset of heat stress and quantify the magnitude of the loss in production and in health performances for Montbeliarde cows; and (2) to estimate genetic-by-THI interactions, which will enable the identification of the animals that are most adapted for coping with the expected rises in temperatures.

## Methods

### Dataset

The performance and pedigree information used in this study are from the French national database. Data from first (L1) and second (L2) lactation test-day records on Montbeliarde cows were selected from five French regions (Franche-Comté, Rhône-Alpes, Auvergne, Bretagne and Pays-de-Loire) over a period of four years (August 2016 to June 2020). Six phenotypes were analyzed: milk yield (MY), fat content (FC), protein content (PC), fat yield (FY), protein yield (PY), and somatic cell score (SCS). SCS was obtained as a log- transformation of somatic cell concentration (SCS = log2(SCC/100,000) + 3) [17]. All the phenotypes were monthly recorded. Only animals with known parents, and age at calving between 23 and 42 months at first parity and between 35 and 60 months at second parity were considered. Records on lactations that lasted less than 120 days were removed, as well as all test-day records after 305 days in milk (DIM). Data from herd-year-parity (HY) combinations with less than five cows and herd-test-day-parity (HTD) combinations with less than three records were discarded. Finally, about 25 and 30% of herds were randomly sampled in L1 and L2, respectively, which resulted in a reduced dataset that was more computationally tractable for REML variance components estimation. The final data on first parity comprised 446,717 test-day records from 55,650 cows, and the final data on second parity comprised 457,516 test-day records from 58,229 cows. The pedigree of these animals was traced back to three generations. After trimming, the L1 and L2 pedigree files included 132,562 and 143,008 animals, respectively. Statistics on raw data are in Table 1.

**Table 1:** Number of test-day records, number of cows and means and standard deviations (SD, in parentheses) for milk yield, fat and protein contents, fat and protein yields and somatic cell score by parity

Lactation rank	Number of records	Number of cows	MY (kg/d)	FC (‰)	PC (‰)	FY (g/d)	PY (g/d)	SCS
Lactation 1	446,717	55,650	21.2 (5.1)	38.6 (5.2)	33.3 (3.0)	811 (202)	702 (171)	2.41 (1.68)
Lactation 2	457,516	58,229	24.4 (6.7)	38.8 (5.7)	33.6 (3.3)	938 (259)	813 (213)	2.23 (1.79)

*MY* milk yield, *FY* fat yield, *PY* protein yield, *FC* fat content, *PY* protein content, *SCS* somatic cell score

The meteorological SAFRAN data provided by the French national meteorological agency (Météo-France) included daily estimates of various indicators for each of the 9892 8 × 8-km^2^ squares covering the whole French territory. The use of modeled data instead of observed data was proposed by Mbuthia et al. [18]. These daily estimates are the result from a model developed for meteorological forecasting that uses local recordings that are obtained continuously over a wide network of meteorological stations. The meteorological variables used in this study were average daily temperature and relative humidity. The daily meteorological data assigned to each herd (geographically identified by its zipcode) were weighted averages of the values at each of the 8 × 8-km^2^ squares that covered the location, with the weights equal to the proportion of the surface of the village in each square. The daily THI was calculated as THI = (1.8 × T + 32)-(0.55–0.0055 × RH) × (1.8 × T-26) [15], where T is the 24 h-average temperature (°C), and RH is the 24 h-average relative humidity (%) as used in Bohlouli et al. [19] and Brügemann et al. [20].

In a preliminary investigation, different lengths of THI were tested by considering either only the date of the test-day or the average of the THI at the test-day and from one to six days before, totalizing a range of one to seven days of potential heat stress. It was concluded that the average THI over three days (i.e. at the date of the test-day and the two previous days), was optimal to observe the most marked effects of heat stress. Similar results were obtained by Carabaño et al. [21] and Santana et al. [22]. Although a longer period would better account for a lagged or accumulated effect of extreme THI values, longer periods may also average more variable conditions, thus minimizing the most extreme values.

### Models used for the analyses

The estimate of the average THI effect on milk production performances was obtained with the following model including THI as a fixed effect:

1 $$\mathbf{y}=\mathbf{X}{\boldsymbol{\upbeta}}+\mathbf{Z}\mathbf{a}+\mathbf{W}\mathbf{p}+\mathbf{e},$$

where $\mathbf{y}$ is the vector of the observed performance for a given trait; ${\boldsymbol{\upbeta}}$ is the vector of fixed effects including: (i) herd-year combinations (5686 levels for L1; 7445 levels for L2), (ii) days in milk (DIM) defined by 10d-classes (31 levels), (iii) age at calving defined by months (20 levels in L1, 26 levels in L2), (iv) days carried calf (days from successful insemination until test-day, converted to months, eight levels), (v) month of calving (12 levels), and (vi) the factor of interest, THI, rounded to the nearest integer number and treated as classes, with the first class pooling all THI ≤ 21, and the last class all THI ≥ 77, totalizing 57 levels; $\mathbf{a} \sim N\left(\mathbf{0},\mathbf{A}\,{\upsigma }_{\mathrm{a}}^{2}\right)$ and $\mathbf{p} \sim N\left(\mathbf{0},\mathbf{I}\,{\upsigma }_{\mathrm{p}}^{2}\right)$ are the vectors of random additive genetic and permanent environment effects, respectively; $\mathbf{X}$, $\mathbf{Z}$, and $\mathbf{W}$ are the corresponding incidence matrices; and $\mathbf{e} \sim N\left(\mathbf{0},\mathbf{I}\,{\upsigma }_{\mathrm{e}}^{2}\right)$ is the vector of random residuals.

It should be noted that, in this model, the contemporary groups were not defined by HTD, to avoid capturing all the effects of the THI. For this reason, the HY effect was preferred as a contemporary group. Although it may be argued that the effects of month of calving probably capture some of the THI effects, ignoring seasonal variations (photoperiod, diet, etc.) are more likely to inflate the estimates of THI effects, and therefore month of calving was also kept as a fixed effect.

To estimate genetic-by-THI interactions, random regression models were fitted. Random regression models allow to predict the performance of each genotype under a given environmental condition. HTD was considered as the contemporary group in this model because although it captures the average THI effect, it does not affect the comparison between cows. Since the six traits that were evaluated in this study are known to vary with DIM, the model included random regressions on both THI and DIM as follows:

2 $$\mathbf{y}={\mathbf{X}}_{\mathbf{r}}{{\boldsymbol{\upbeta}}}_{\mathbf{r}}+{\mathbf{Z}}_{\mathbf{r}}{\mathbf{a}}_{\mathbf{r}}+{\mathbf{W}}_{\mathbf{r}}{\mathbf{p}}_{\mathbf{r}}+{\mathbf{e}}_{\mathbf{r}},$$

where ${{\boldsymbol{\upbeta}}}_{\mathbf{r}}$ is the vector of fixed effects including herd-test-day as a contemporary group (45,118 levels in L1; 57,022 levels in L2) and days in milk, age at calving, and days carried calf as defined in Model (1). The additive genetic effect ${\mathbf{a}}_{\mathbf{r}}$ was regressed with six functions: an intercept independent of DIM and THI, two DIM-dependent functions (linear and quadratic functions) and three THI-dependent functions (linear, quadratic and cubic functions). For computational reasons, only two functions were considered for DIM. All random regressions were modeled with Legendre polynomials. ${\mathbf{Z}}_{\mathbf{r}}$ is a known matrix of covariates, with six non-zero terms on each line, equal to a constant, the first and second order Legendre polynomials of DIM, and the first, second, and third order Legendre polynomials of THI, respectively. ${\mathbf{a}}_{\mathbf{r}}$ is the corresponding vector of animal additive genetic regression coefficients, with six values per animal. The arguments of the polynomials were standardized values of THI and DIM within the − 1 to + 1 interval. For DIM, extreme values were 7 and 305 days, and 0 corresponded to 156 days. For THI, extreme values were 18 and 78, with 0 corresponding to THI 48. The same random regressions were applied to the permanent environmental effect. Therefore, ${\mathbf{W}}_{\mathbf{r}}$ is similar to ${\mathbf{Z}}_{\mathbf{r}}$ but restricted to animals with records and ${\mathbf{p}}_{\mathbf{r}}$ is the corresponding vector of permanent environment regression coefficients, with six values per animal with records. Finally, ${\mathbf{e}}_{\mathbf{r}}$ is the vector of independent residuals, with heterogeneous variances across 55 classes created by combining 11 DIM classes (7-14d, 15-29d, 30-59d, 60-89d, 90-119d, 120-149d, 150-179d, 180-209d, 210-239d, 240-269d, 270-305d) with five THI classes (≤ 29, 30–39, 40–49, 50–59,  ≥ 60). These classes were defined based on preliminary analyses and we chose to keep evenly spaced intervals even if the differences between some adjacent classes were small.

The genetic random regression variance matrix $\mathrm{var}({\mathbf{a}}_{\mathrm{r}})=\mathbf{G}$ is a 6-by-6 symmetric matrix:

$$\mathbf{G}=\left[\begin{array}{cccccc}{\upsigma }_{{\mathrm{a}}_{1}}^{2}& {\upsigma }_{{\mathrm{a}}_{1}\_{\mathrm{a}}_{2}}& {\upsigma }_{{\mathrm{a}}_{1}\_{\mathrm{a}}_{3}}& {\upsigma }_{{\mathrm{a}}_{1}\_{\mathrm{a}}_{4}}& {\upsigma }_{{\mathrm{a}}_{1}\_{\mathrm{a}}_{5}}& {\upsigma }_{{\mathrm{a}}_{1}\_{\mathrm{a}}_{6}}\\ .& {\upsigma }_{{\mathrm{a}}_{2}}^{2}& {\upsigma }_{{\mathrm{a}}_{2}\_{\mathrm{a}}_{3}}& {\upsigma }_{{\mathrm{a}}_{2}\_{\mathrm{a}}_{4}}& {\upsigma }_{{\mathrm{a}}_{2}\_{\mathrm{a}}_{5}}& {\upsigma }_{{\mathrm{a}}_{2}\_{\mathrm{a}}_{6}}\\ .& .& {\upsigma }_{{\mathrm{a}}_{3}}^{2}& {\upsigma }_{{\mathrm{a}}_{3}\_{\mathrm{a}}_{4}}& {\upsigma }_{{\mathrm{a}}_{3}\_{\mathrm{a}}_{5}}& {\upsigma }_{{\mathrm{a}}_{3}\_{\mathrm{a}}_{6}}\\ .& .& .& {\upsigma }_{{\mathrm{a}}_{4}}^{2}& {\upsigma }_{{\mathrm{a}}_{4}\_{\mathrm{a}}_{5}}& {\upsigma }_{{\mathrm{a}}_{4}\_{\mathrm{a}}_{6}}\\ .& .& .& .& {\upsigma }_{{\mathrm{a}}_{5}}^{2}& {\upsigma }_{{\mathrm{a}}_{5}\_{\mathrm{a}}_{6}}\\ .& .& .& .& .& {\upsigma }_{{\mathrm{a}}_{6}}^{2}\end{array}\right],$$

where ${\upsigma }_{{\mathrm{a}}_{\mathrm{i}}}^{2}$ is the variance of the $i$th regression coefficient of Legendre polynomial, ${\upsigma }_{{\mathrm{a}}_{\mathrm{i}}\_{\mathrm{a}}_{\mathrm{j}}}$ is the covariance between coefficients $i$ and $j$ of the regression.

The genetic variances and covariances at each DIM and each THI were estimated by pre- and post-multiplying the variance matrix $\mathbf{G}$ by the corresponding DIM- and THI-coefficients of Legendre polynomials using the formulas:

3 $${\mathrm{varG}}_{({\mathrm{d}}_{1},{\mathrm{t}}_{1})}={\mathbf{z}}_{({\mathrm{d}}_{1},{\mathrm{t}}_{1})} \mathbf{G}\,{ \mathbf{z}}_{t({\mathrm{d}}_{1},{\mathrm{t}}_{1})}^{\mathrm{{\prime}}},$$

4 $${\mathrm{covG}}_{({\mathrm{d}}_{1},{\mathrm{t}}_{1} ; {\mathrm{d}}_{2}{\mathrm{t}}_{2})}={\mathbf{z}}_{({\mathrm{d}}_{1},{\mathrm{t}}_{1})} \mathbf{G}\,{ \mathbf{z}}_{({\mathrm{d}}_{2},{\mathrm{t}}_{2})}^{\mathrm{{\prime}}},$$

where ${\mathrm{varG}}_{({\mathrm{d}}_{1},{\mathrm{t}}_{1})}$ is the genetic variance evaluated at DIM ${d}_{1}$ and THI ${t}_{1}$; ${\mathrm{covG}}_{({\mathrm{d}}_{1},{\mathrm{t}}_{1} ; {\mathrm{d}}_{2}{\mathrm{t}}_{2})}$ is the genetic covariance between the combination of DIM ${d}_{1}$ and THI ${t}_{1}$ and the combination of DIM ${d}_{2}$ and THI ${t}_{2}$; ${\mathbf{z}}_{({d}_{1},{t}_{1})}$ is the vector of covariates estimated at DIM ${d}_{1}$ and THI ${t}_{1}$ and ${\mathbf{z}}_{({d}_{2},{t}_{2})}$ is the vector of covariates estimated at DIM ${d}_{2}$ and THI ${t}_{2}$.

The permanent environmental random regression variance matrix and the permanent environmental variances were defined similarly as follows:

5 $${\mathrm{varP}}_{({\mathrm{d}}_{1},{\mathrm{t}}_{1})}={\mathbf{z}}_{({\mathrm{d}}_{1},{\mathrm{t}}_{1})} \mathbf{P}\,{ \mathbf{z}}_{({\mathrm{d}}_{1},{\mathrm{t}}_{1})}^{\mathrm{{\prime}}},$$

with $\mathbf{P}$ being the 6-by-6 symmetric permanent environmental random regression variance matrix.

The residual variance ${\mathrm{varR}}_{({\mathrm{d}}_{1},{\mathrm{t}}_{1})}$ is the residual variance estimated at DIM ${d}_{1}$ and THI ${t}_{1}$.

All these variances allowed to calculate the heritabilities at DIM ${d}_{1}$ and THI ${t}_{1}$ from:

6 $${\mathrm{h}}_{({\mathrm{d}}_{1},{\mathrm{t}}_{1})}^{2}=\frac{{\mathrm{varG}}_{({\mathrm{d}}_{1},{\mathrm{t}}_{1})}}{{\mathrm{varG}}_{({\mathrm{d}}_{1},{\mathrm{t}}_{1})}+{\mathrm{varP}}_{({\mathrm{d}}_{1},{\mathrm{t}}_{1})}+{\mathrm{varR}}_{({\mathrm{d}}_{1},{\mathrm{t}}_{1})}}$$

and the correlations between traits across DIM and THI as follows:

7 $${\mathrm{r}}_{{\mathrm{g}}_{({\mathrm{d}}_{1},{\mathrm{t}}_{1} ; {\mathrm{d}}_{2},{\mathrm{t}}_{2})}}=\frac{{\mathrm{covG}}_{({\mathrm{d}}_{1},{\mathrm{t}}_{1} ; {\mathrm{d}}_{2},{\mathrm{t}}_{2})}}{\sqrt{{\mathrm{varG}}_{({\mathrm{d}}_{1},{\mathrm{t}}_{1})}*{\mathrm{varG}}_{({\mathrm{d}}_{2},{\mathrm{t}}_{2})}}},$$

where ${\mathrm{r}}_{{\mathrm{g}}_{({\mathrm{d}}_{1},{\mathrm{t}}_{1} ; {\mathrm{d}}_{2},{\mathrm{t}}_{2})}}$ is the genetic correlation between the trait at DIM ${d}_{1}$ and THI ${t}_{1}$ and the same trait at DIM ${d}_{2}$ and THI ${t}_{2}$.

Finally, this random regression model defined in Eq. (2) allows to estimate breeding values along the DIM and the THI gradients as:

8 $${\mathrm{EBV}}_{\mathrm{i}\_{\mathrm{d}}_{1}{\mathrm{t}}_{1}}={\mathbf{z}}_{({\mathrm{d}}_{1},{\mathrm{t}}_{1} )}{\widehat{\mathbf{a}}}_{\mathrm{ri}},$$

where ${\mathrm{EBV}}_{\mathrm{i}\_{\mathrm{d}}_{1}{\mathrm{t}}_{1}}$ is the estimated breeding value for the $i$th animal at DIM ${d}_{1}$ and THI ${t}_{1}$; ${\widehat{\mathbf{a}}}_{\mathrm{ri}}$ is the vector of the six random regression coefficients estimated for the $i$th animal.

The slope of the regression on (standardized) THI for animal $i$ was computed using the following derivative formula:

9 $$\frac{\mathrm{d}\mathbf{z}}{\mathrm{dTHI}}{\mathrm{a}}_{\mathrm{r}}\left(\mathrm{i}\right)=\frac{{\mathrm{dz}}_{4}}{\mathrm{dTHI}}{\mathrm{a}}_{{\mathrm{r}}_{4}}\left(\mathrm{i}\right)+ \frac{{\mathrm{dz}}_{5}}{\mathrm{dTHI}}{\mathrm{a}}_{{\mathrm{r}}_{5}}\left(\mathrm{i}\right)+ \frac{{\mathrm{dz}}_{6}}{\mathrm{dTHI}}{\mathrm{a}}_{{\mathrm{r}}_{6}}\left(\mathrm{i}\right).$$

10 $$\mathrm{Its\,variance\,was\,computed\,as\,var}{\mathrm{G}}_{(\mathrm{slope})= }\frac{\mathrm{d}\mathbf{z}}{\mathrm{dTHI}}\mathbf{G} {\left(\frac{\mathrm{d}\mathbf{z}}{\mathrm{dTHI}}\right)}^{{\prime}}.$$

The genetic correlation between breeding value at DIM ${d}_{1}$ and THI ${t}_{1}$ and the slope of the regression on THI can be calculated as follows:

11 $${\mathrm{r}}_{{\mathrm{g}}_{({\mathrm{d}}_{1},{\mathrm{t}}_{1} ;\mathrm{ slope})}}=\frac{{\mathrm{covG}}_{({\mathrm{d}}_{1},{\mathrm{t}}_{1} ;\mathrm{ slope})}}{\sqrt{{\mathrm{varG}}_{({\mathrm{d}}_{1},{\mathrm{t}}_{1})}*{\mathrm{varG}}_{(\mathrm{slope})}}},$$

12 $${\mathrm{with\,covG}}_{({\mathrm{d}}_{1},{\mathrm{t}}_{1} ;\mathrm{ slope})}= {\mathbf{z}}_{({\mathrm{d}}_{1},{\mathrm{t}}_{1})} \mathbf{G}{ \left(\frac{\mathrm{d}\mathbf{z}}{\mathrm{dTHI}}\right)}^{{\prime}}.$$

In all the analyses, the six traits (MY, FC, PC, FY, PY and SCS) were evaluated separately. All fixed effects and variance components were estimated using the WOMBAT software [23].

## Results

### Climate conditions

**Fig. 1:**
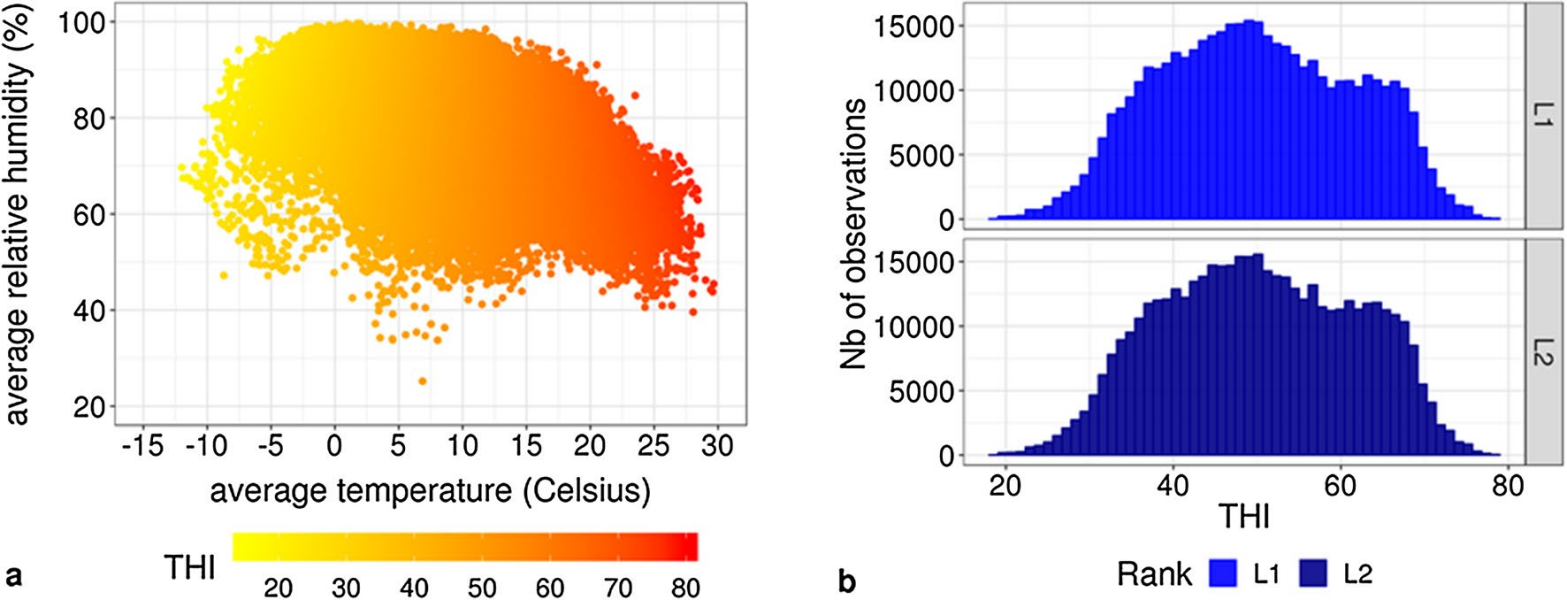
Descriptive analysis of meteorological variables. **a** Observed average temperatures (°C) by relative humidity (%), with the corresponding color-coded temperature-humidity index (THI). **b** Histogram of the distribution of the observed THI in first (L1) and second (L2) lactation

Figure 1 provides a descriptive analysis of the meteorological variables observed for the herds during the period of time when our studied test-day records were collected. The 3d-average temperatures ranged from − 12 to + 30 °C, with moderate humidity levels when temperatures w ere high (Fig. 1a). THI ranged from 18 to 78, with only 3.2 and 3.1% of the test-day performances recorded at THI ≥ 70 during L1 and L2, respectively.

### Effect of THI on production and udder health traits

**Fig. 2:**
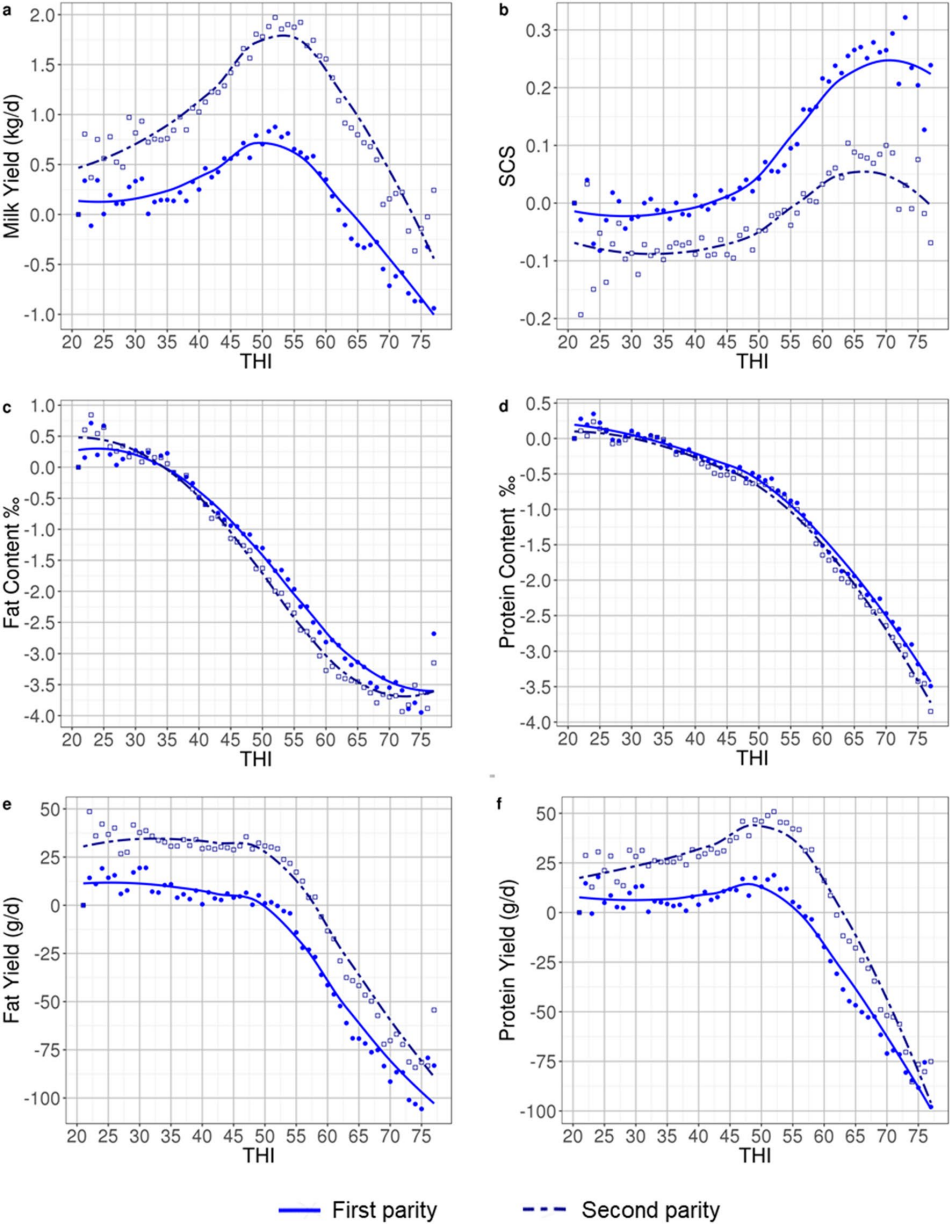
Average effects of THI in **a** milk yield; **b** somatic cell score (SCS); **c** fat and **d** protein contents; **e** fat and **f** protein yields in first (solid line) and second (dashed line) lactation

The average effect of THI on performances, according to the model in Eq. (1) is shown in Fig. 2. The optimal THI, determined as the THI allowing for the best level of performance, differed across traits but was similar for the two parities within each trait. While all six traits showed a visible detrimental effect of heat stress, an effect of cold stress was only observed for MY and PY, although it was less pronounced than that of heat stress, especially for PY in both L1 and L2 and MY in L1. In L2, the optimal THI was 50–55 (i.e. 7 to 13 °C) for both MY and PY, while in L1 the optimal THI seemed slightly lower and around 50 for these two traits. Both FC and PC presented a decline for THI > 35 (i.e. above the range from − 2.5 to + 2 °C), FY presents a decline for THI > 50 (i.e. above the range from 7 to 10 °C), and SCS presented a detrimental effect of THI > 45 (i.e. above the range from 3 to 7 °C). The estimates of average losses between performances recorded under optimal THI and performances recorded under the most unfavorable heat stress condition with THI ≥ 70 are in Table 2 and show a very similar impact of heat stress in both parities for production traits. The average relative loss was lowest (~ 7%, i.e. − 0.07 kg.d^−1^ and − 0.09 kg.d^−1^ per unit of additional THI in L1 and L2, respectively) for MY, and was highest (~ 11.5–13.5%, i.e. around − 5 g.d^−1^ per unit of additional THI) for FY and PY. The effect of heat stress on SCS was stronger in L1 (average increase of 10.3%) than in L2 (average increase of 4.8%).

**Table 2:** Difference between averages of adjusted performances recorded at the optimal temperature-humidity index (THI) and averages of adjusted performances recorded under the strongest heat stress conditions (THI ≥ 70) and slopes above optimal THI (per unit of additional THI)

	MY	FC	PC	FY	PY	SCS
Optimal THI						
L1	50–55	≤ 35	≤ 35	≤ 50	45–55	≤ 45
L2	50–55	≤ 35	≤ 35	≤ 50	45–55	≤ 45
Average absolute differences between performances recorded at optimal THI and at THI ≥ 70						
L1	− 1.49 kg/d	− 3.82‰	− 3.04‰	− 100.4 g/d	− 92.9 g/d	+ 0.25
L2	− 1.86 kg/d	− 4.00‰	− 3.23‰	− 107.8 g/d	− 112.5 g/d	+ 0.11
Average relative differences between performances recorded at optimal THI and at THI ≥ 70 (% overall mean)						
L1	− 7.0	− 9.9	− 9.1	− 12.4	− 13.2	+ 10.3
L2	− 7.4	− 10.3	− 9.6	− 11.5	− 13.8	+ 4.8
Average slopes above optimal THI						
L1	− 0.07 kg/d	− 0.11‰	− 0.09‰	− 5.02 g/d	− 4.64 g/d	+ 0.010
L2	− 0.09 kg/d	− 0.11‰	− 0.09‰	− 5.39 g/d	− 5.63 g/d	+ 0.004

*MY* milk yield, *FY* fat yield, *PY* protein yield, *FC* fat content, *PY* protein content, *SCS* somatic cell score, *L1* = first lactation, *L2* second lactation

### Genetic-by-THI interactions

**Fig. 3:**
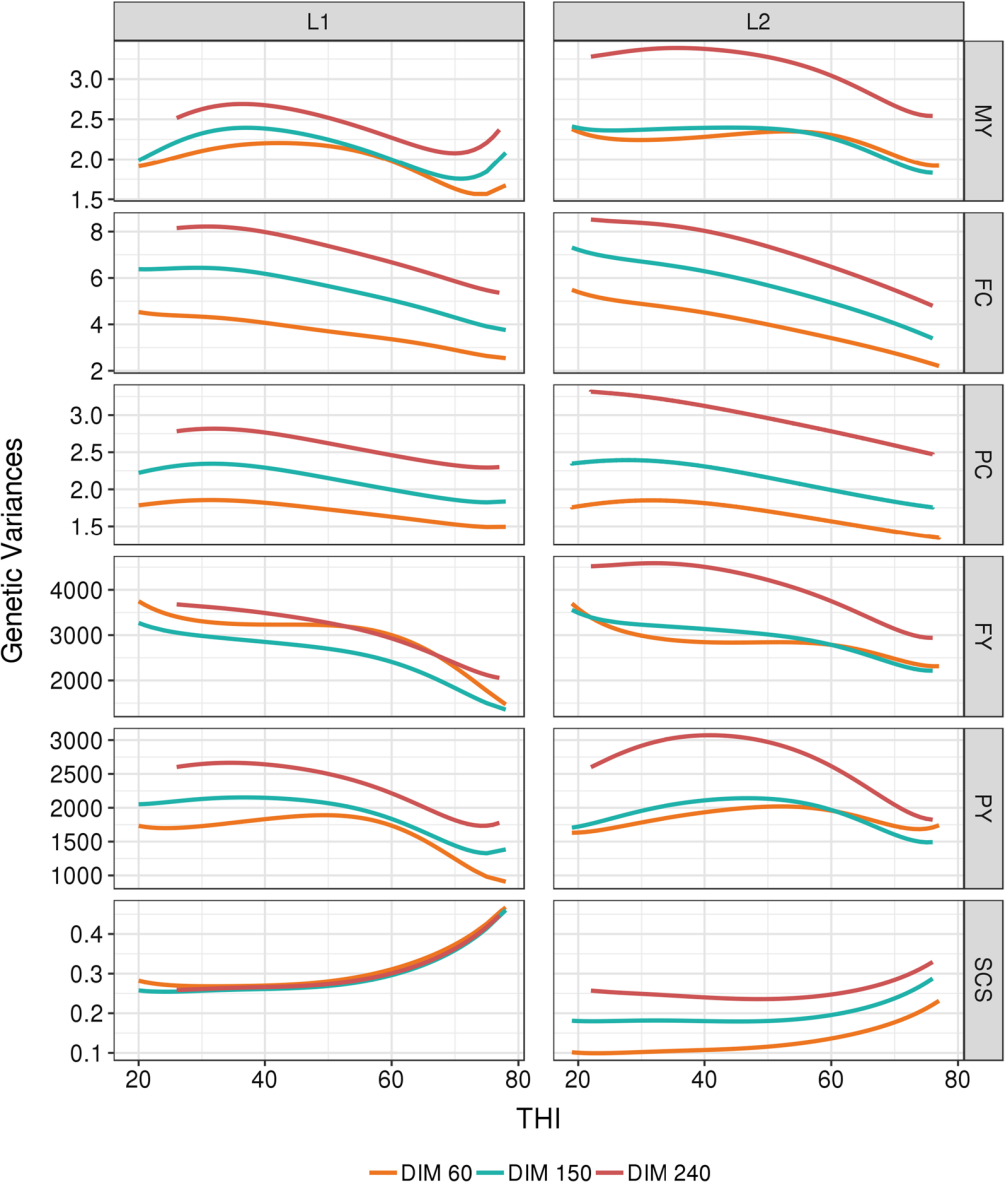
Evolution of additive genetic variances for each trait with THI at three selected days-in-milk (DIM) in first (L1) and second (L2) lactation. For all traits and both parities, the standard errors range from 4 to 15%, 4 to 30%, and 5 to 22% of the estimated genetic variances when the THI is between 30 and 70, below 30, and above 70, respectively. *MY* milk yield, kg^2^, *FY* fat yield, kg^2^, *PY* protein yield, kg^2^, *FC* fat content, (g/kg)^2^, *PY* protein content, (g/kg)^2^, *SCS* somatic cell score

**Fig. 4:**
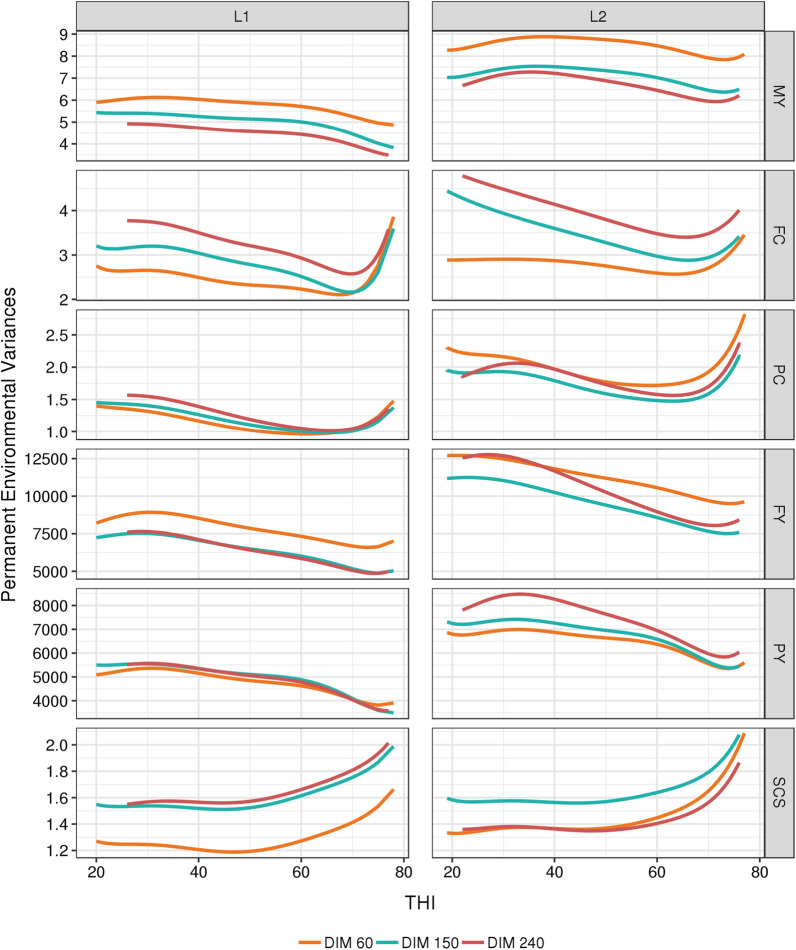
Evolution of permanent environmental variances for each trait with THI at three selected days-in-milk (DIM) in first (L1) and second (L2) lactation. *MY* milk yield, kg^2^, *FY* fat yield, kg^2^, *PY* protein yield, kg^2^, *FC* fat content, (g/kg)^2^, *PY* protein content, (g/kg)^2^, *SCS* somatic cell score

**Fig. 5:**
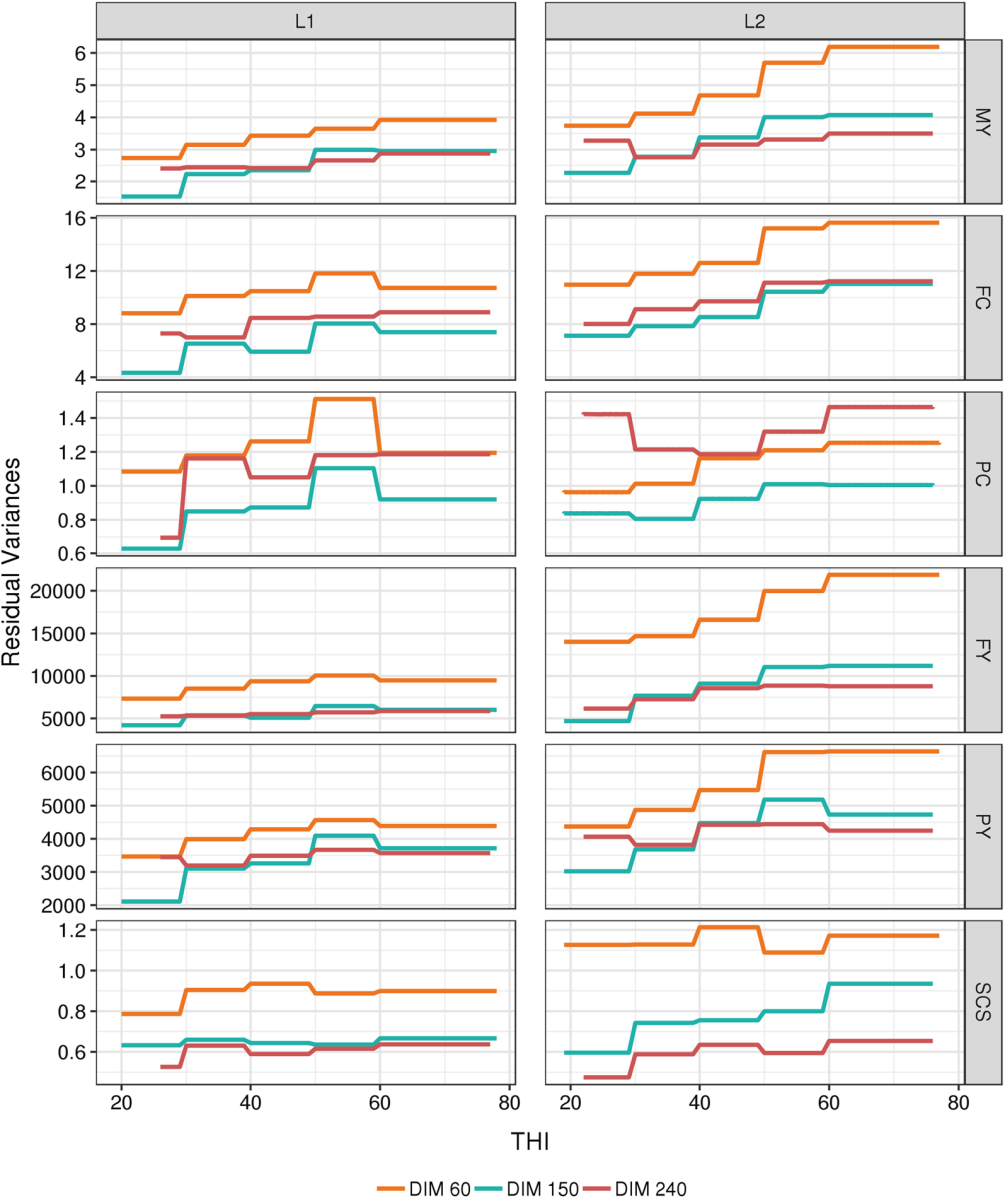
Evolution of residual variances for each trait with THI at three selected days-in-milk (DIM) in first (L1) and second (L2) lactation. *MY* milk yield, kg^2^, *FY* fat yield, kg^2^, *PY* protein yield, kg^2^, *FC* fat content, (g/kg)^2^, *PY* protein content, (g/kg)^2^, *SCS* somatic cell score

With the random regression models used, different variances across all combinations of DIM and THI were obtained for each trait. Since the main objective of this study was to investigate tolerance to heat stress in dairy cows, all the estimates of variance components, heritabilities, and correlations or breeding values are illustrated along the THI gradient, and at three key stages of lactation: (1) at 60d, just after the peak of lactation; (2) at 150d, in the middle of the lactation curve; and (3) at 240d, when production is also affected by persistency. Even if the shapes of the curves were not identical, for all production traits and in both lactations, Fig. 3 shows that the trends were quite general with the largest additive genetic variances for the lowest THI (i.e. THI < 40) and a decrease in additive genetic variances along the THI gradient. PY was the only production trait with a mildly outstanding pattern, presenting the largest additive genetic variances between a THI of 40 and 60 up to 120 DIM in L1 and up to 210 DIM in L2 (see Additional file 1: Fig. S1). In contrast to the production traits, the largest genetic variances for SCS were obtained for the highest THI (i.e. THI > 60). Similar to the genetic variances, the permanent environmental variances also tended to decrease with increasing THI for all production traits and to increase with higher THI for SCS (Fig. 4). However, for PC and FC, we observed a major difference, i.e. at the highest THI, a large rebound was observed in the values of the permanent environmental variances. Residual variances for all traits increased with increasing THI for both production and udder health traits (Fig. 5).

**Fig. 6:**
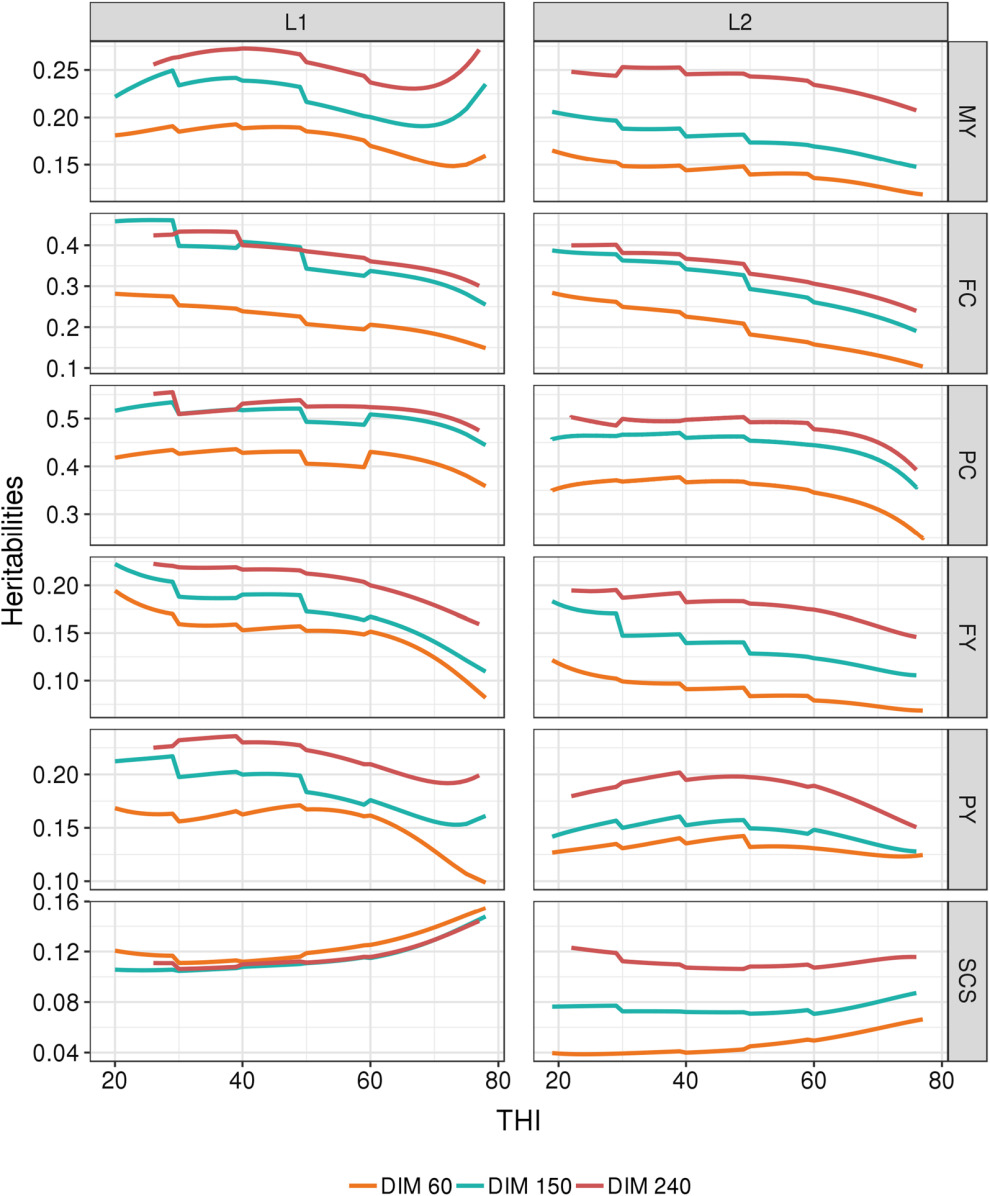
Trajectories of estimates of heritability for each trait with THI at three selected days-in-milk (DIM) in first (L1) and second (L2) lactation. For all traits and both parities, the standard errors range from 4 to 15%, 4 to 30%, and 4 to 22% of the estimated genetic variances when the THI is between 30 and 70, below 30, and above 70, respectively. *MY* milk yield, kg^2^, *FY* fat yield, kg^2^, *PY* protein yield, kg^2^, *FC* fat content, (g/kg)^2^, *PY* protein content, (g/kg)^2^, *SCS* somatic cell score

For production traits, the patterns of the change in heritability with THI (see Fig. 6) reflected those for the genetic variances with the heritability tending to decrease a little with increasing THI. However, for PC and PY, this decrease in heritability occurred only for the highest THI values. For SCS, the trend in heritability along the THI gradient differed between parities, with an equivalent increase in heritability with increasing THI at all DIM in L1, while in L2 this increase in heritability was lower and concentrated at the early stages of lactation. Among the six traits evaluated, SCS was the only one to show a marked difference in the trends of heritability through THI between parities.

**Fig. 7:**
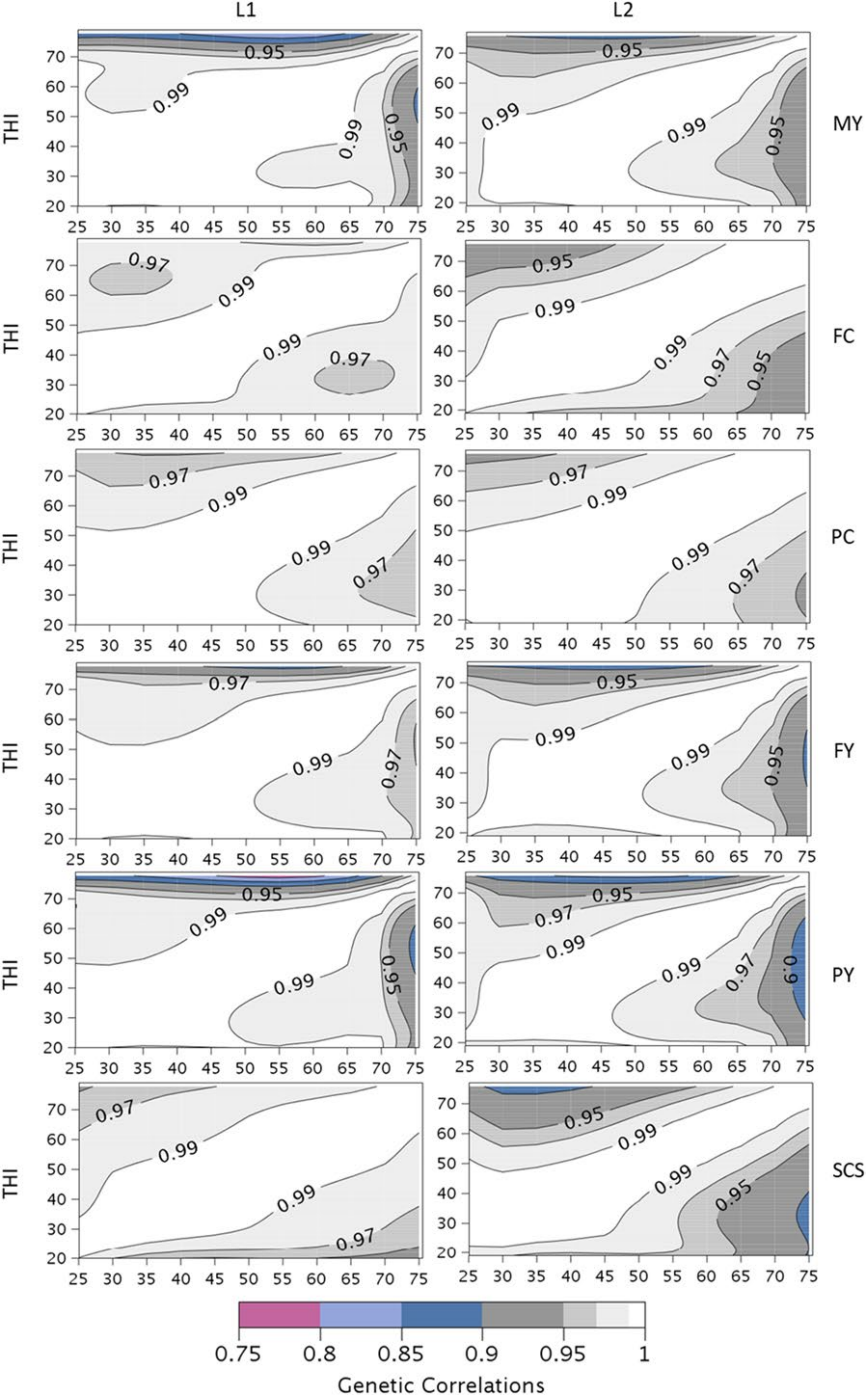
Estimates of genetic correlations within trait at different THI for milk yield (MY), somatic cell score (SCS), fat and protein contents (FC and PC) and fat and protein yields (FY and PY) in first (L1) and second (L2) lactation. Results are given at 150 days-in-milk. Standard errors range from 0 to 0.15

Most of the estimated genetic correlations between different THI values, within trait, parity and DIM, were higher than 0.90, as shown in Fig. 7. For FC, PC and SCS in L1, none of the estimated genetic correlations were lower than 0.90. Overall, the lowest correlations were rarely lower than 0.80, and these were always correlations of the highest THI, above 70, with distant THI. The lowest genetic correlations were obtained for PY, in both L1 and L2, but they remained above 0.75. For all traits, the estimated genetic correlation between the neutral THI of 50 and the heat stress THI of 70 were lower in L2 than in L1, and in most cases, it reached the lowest values just after the lactation peak (DIM = 60d) and tended to increase with DIM, as shown in Table 3.

**Table 3:** Genetic correlations from random regression model analyses for MY, FC, PC, FY, PY, and SCS between THI = 50 and THI = 70 at different stages of lactation (60d, 150d and 240d) and for lactations L1 and L2

	MY		FC		PC		FY		PY		SCS	
DIM (d)	L1	L2	L1	L2	L1	L2	L1	L2	L1	L2	L1	L2
60	0.970	0.960	0.976	0.957	0.977	0.976	0.985	0.954	0.969	0.945	0.989	0.945
150	0.970	0.962	0.988	0.973	0.983	0.982	0.982	0.959	0.969	0.952	0.988	0.955
240	0.972	0.974	0.991	0.979	0.986	0.987	0.984	0.976	0.975	0.974	0.988	0.961

*MY* milk yield, *FY* fat yield, *PY* protein yield, *FC* fat content, *PY* protein content, *SCS* somatic cell score, *DIM* days in milk, *L1* first lactation, *L2* second lactation

**Fig. 8:**
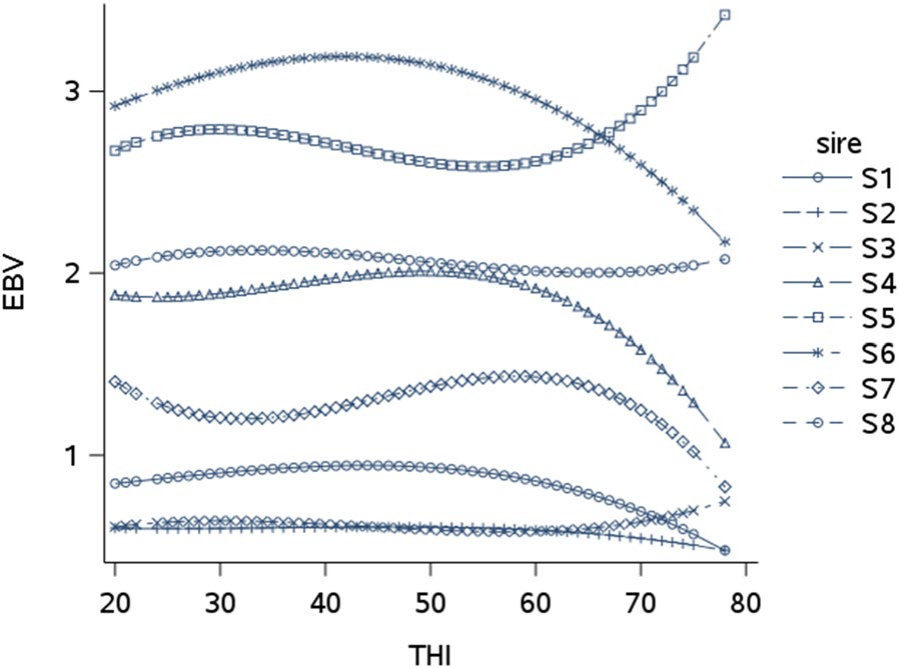
Estimated breeding values (EBV) for milk yield (kg/day) in first lactation for eight sires (S1-S8) with at least 500 daughters with performances. Results are provided at day in milk 150

Although the genetic correlations suggested that the genetic-by-THI interactions were weak, they were not equal to 1, thus we examined the evolution of individual estimated breeding values (EBV) along the THI gradient. Figure 8 illustrates some examples of EBV for milk yield in L1, for some of the most widely used sires in our dataset. These trajectories show that the ranking of sires remained stable between THI 20 and 60 but for THI values greater than 60, reranking occurred, i.e. at high THI, some sires experienced a decrease in EBV while others moved up in the ranking. These changes in ranking of EBV can be quantified by calculating the slopes at a given THI. For instance, at THI 70, the slope of the sire S5 was equal to + 0.048 kg.d^−1^/unit of additional THI and the slopes of the sires S4 and S6 were − 0.050 and − 0.046 kg.d^−1^/unit of additional THI, respectively. The estimates of the genetic standard deviation of the slopes at THI 70 and the total genetic standard deviation of the trait at THI 70 are in Table 4 and show that for all traits, the genetic variability of the slope remained very low compared to the total genetic variability of the trait at THI 70. The highest proportion was estimated at 5% of the total genetic variance for PY at THI = 70 (5.17 and 5.09% in L1 and L2, respectively). These effects expressed in kg per day represent a limited loss in milk quantity if the hot period is short. However, if it is assumed that these estimates also stand for longer periods and that such hot conditions will last for 4 or 6 months in the future (a situation predicted for a large part of France), this estimate may represent a 0.2 to 0.3 genetic standard deviation over the whole lactation and cannot be neglected. Thus, it was relevant to estimate the genetic correlations between these slopes at high THI and the breeding values at moderate THI, to predict the consequences on these slopes of the current selection and assess if high-producing animals are less or more affected than the others. These estimates of genetic correlations between slopes at high THI and total breeding value at medium THI are in Table 4. For the production traits, the correlations were negative, which indicates that the animals with the highest breeding values were those with the most unfavorable slopes, while for SCS, the estimates were positive, which indicates that the susceptible animals in standard conditions were even more susceptible in heat stress conditions. These correlations were moderate (around − 0.3 for MY and PC in L1 and + 0.33 for SCS in L2) to high (− 0.87 for FC in L1).

**Table 4:** Genetic standard deviations of slopes at a temperature-humidity index (THI) of 70, genetic standard deviations of the trait at a THI of 70 and days in milk (DIM) = 150d and genetic correlations between slopes at a THI of 70 and breeding value at a THI of 50 and DIM = 150d

	MY	FC	PC	FY	PY	SCS
Genetic standard deviation of slope at THI = 70 (per unit of additional THI)						
L1	0.051 kg/d	0.038‰	0.023‰	1.395 g/d	1.576 g/d	0.011
L2	0.047 kg/d	0.034‰	0.018‰	1.719 g/d	1.649 g/d	0.012
Genetic standard deviation of the trait at THI = 70 and DIM = 150d						
L1	1.33 kg/d	2.07‰	1.36‰	42.81 g/d	37.96 g/d	0.599
L2	1.40 kg/d	2.01‰	1.35‰	48.67 g/d	40.04 g/d	0.487
Genetic correlation between slope at THI = 70 and breeding value at THI = 50 and DIM = 150d						
L1	− 0.27	− 0.53	− 0.29	− 0.67	− 0.50	+ 0.64
L2	− 0.46	− 0.87	− 0.45	− 0.47	− 0.51	+ 0.33

*MY* milk yield, *FY* fat yield, *PY* protein yield, *FC* fat content, *PY* protein content, *SCS* somatic cell score, *L1* first lactation, *L2* second lactation

## Discussion

### Effect of THI on production and udder health traits

The aim of our study was to estimate the effect of THI on production and udder health traits in Montbeliarde dairy cattle, and to identify THI values associated with the onset of heat stress for the aforementioned traits. To our knowledge, this is the first study of heat stress in this breed, as well as the first one to account for daily THI information on French bovine data, regardless of the breed. Therefore, we compared our results to those obtained on other high yielding breeds that are known to be less adapted to heat than tropical breeds (see review in [24]. In the literature, there are large differences in the thresholds of heat stress among breeds [9, 16, 25], climatic indices used [13], traits studied [21, 25–29] and climatic regions [21], and it is important to keep this in mind when comparing our results to those previously reported.

Our results highlight a detrimental effect of heat stress on MY for THI > 55, a threshold that is substantially lower than that generally reported in the literature. Indeed, for Holstein cattle, the onset of the decline in MY has been reported to occur at THI between 70 and 78 in Italy, depending on parity [26], at THI of 73 in Spain [21], 69 in Belgium [21], and 60 to 62 in Germany [20]. The results for American Holstein are in line with those for Mediterranean Holstein (Italy or Spain), with MY decreasing from a THI of 72 in the state of Georgia and from a THI of 74 in the state of Arizona [13]. These results on Holstein can be taken as indicators of the thresholds for high-producing dairy cattle. However as previously mentioned, it is important to keep in mind that thresholds vary among breeds, and thus that the results for Holstein may not be completely transposable to the Montbeliarde breed. Indeed, in a study on New Zealand dairy cattle, Bryant et al. [25] highlighted differences in their estimates of thresholds for MY that changed according to the breed analysed within the same geographical regions: THI of 64 in Holstein *vs* THI of 73 in Jersey cattle.

The THI thresholds for the decline in FC, PC, FY, and PY are below the threshold of 55 that was found for MY. On the one hand, this trend towards lower thresholds for milk components is in agreement with the results of Bryant et al. [25] and Carabanõ et al. [21, 28], while on the other hand, Bernabucci et al. [26] and Hammami et al. [14] reported identical THI thresholds for milk, fat and protein yields.

In spite of these differences in THI thresholds, studies on animals raised in countries with a warmer climate than France, and on other breeds than the Montbeliarde, have reported the same trends of the response to THI for fat and protein traits. For example, in Italian Holstein [26] and New Zealand Holstein [25], FC and PC decrease continuously as THI increases, whereas FY and PY remain on a plateau until the THI threshold for the onset of the heat stress is reached and then decline steeply.

Hammami et al. [14] on Luxemburg Holstein, and Hagiya et al. [30] and Ishida et al. [31] on Japanese Holstein reported THI thresholds ranging from 60 to 66 for SCS, which are higher than the value of 45 observed in our study. Moreover, both Hammami et al. [14] and Brügemann et al. [20] indicated an effect of a cold stress on SCS, which was not observed in our study on the Montbeliarde breed.

Several factors may explain the lower THI thresholds observed in our study compared to those commonly reported for high-producing dairy cows. First, many of the studies on high-producing dairy cows concern animals that are raised in indoor farming systems, sometimes in barns equipped with cooling systems, which mitigate the effect of high outdoor temperatures [28, 32]. French Montbeliarde cows are kept outdoors during the spring and summer, and thus are more exposed to warmer temperatures and extreme heat waves, without any mitigation. Second, many studies on the effect of heat stress on high-producing dairy cows are performed in countries with a warmer climate than France and with THI that have higher values than those observed in France for our datasets. For example, in the climate data for some studies, the minimum THI observed was of ~ 50 in Italy [26] and in the United States [33], a value close to our observed THI threshold for several traits. In these countries, a probable acclimatization to warmer temperatures can be suspected. Indeed, in their review, Collier et al. [34] indicate that the chronic response of acclimatization to stress is driven by the continued exposure of the animals to the stressor and can result in a new physiological state. In line with this, comparing Holstein cattle raised in four countries, Belgium, Luxemburg, Slovenia and Spain, Carabaño et al. [21] highlighted that, in most cases, thresholds were higher in Spain than in the other countries and hypothesized a short-term acclimatization explained by a gradual rise in temperatures that remain high along the spring and summer in Spain vs sudden heat waves in Belgium and Luxemburg. Finally, one can also reasonably suggest that these higher threshold values may be due to an indirect selection for heat tolerance. Indeed, since animals are selected within their most frequent production environment, in countries where temperatures are on average higher than in France, the animals that are selected for the highest production are those that are able to produce under warmer conditions.

The rates of the decline in performances that we report here are of the same order of magnitude as those estimated in Carabaño et al. [28]. These authors reported losses per unit of additional °C degree ranging from − 0.09 to − 0.16 kg.d^−1^ for MY, from − 2.9 to − 4.4 g.d^−1^ for FY, − 1.6 to 3.8 g.d^−1^ for PY, and + 0.0027 to + 0.0032 for SCS, depending on the regression approach used to model the effect of THI, splines or Legendre polynomials. Considering the results obtained with the same THI formula as those used in our study, our results support those obtained for animals raised in the crop production system by Brügemann et al. [20], but are lower than the values of − 0.17 and − 0.26 kg.d^−1^ per unit of additional THI that they found for animals raised in the pasture and maritime regions, respectively. Our rates of decline in performances are also of lower intensity than those estimated by Hammami et al. [14] in Luxemburg Holstein for milk, fat and protein yields (− 0.16, − 0.020 and − 0.013 kg.d^−1^ per unit of additional THI, respectively) and by Bohmanova et al. [13] for milk yield (− 0.30 and − 0.39 kg.d^−1^ per unit of additional THI, depending on the USA region investigated).

### Genetic-by-THI interactions

We estimated the trajectory of genetic parameters of production and SCS traits along the THI gradient in Montbeliarde dairy cattle using random regressions and Legendre polynomials. Ravagnolo and Misztal [35] proposed a broken line model to estimate the change in response for MY with THI. Because of the assumption of the presence of a neutral zone with no effect of THI (plateau) followed by a linear decay, the broken line model does not fit our data for which a negative effect of low THI is also found. Moreover, this model requires the estimation of identical thresholds marking the onset of heat stress. However, applying an identical threshold to all the animals, assumes the absence of individual variability in the onset of heat stress, which is contrary to the findings of several authors [33, 36], and could bias the slopes of individual production loss [37]. Although the estimation of an individual threshold is complex [33, 36], Carabaño et al. [28] proposed to use Legendre polynomials across the entire range of THI, without estimating or imposing a threshold. Nevertheless, it is important to highlight that although random regression models with polynomials are suitable, they do have drawbacks. Among these drawbacks, we emphasize the typical border effects at the beginning and the end of the curves, and waves in the middle of a lactation, a misfit of the model that is accentuated when data are scarce [38]. These border effects are especially critical to us, as we are primarily interested in the prediction of the response at high THI conditions.

All the traits studied here have previously been shown to vary genetically with DIM. Time-dependent random regressions allow modelling different lactation curves for each cow [39], thus accounting for genetic variability among cows in lactation onset and persistency. Some general conclusions can be drawn: the beginning of the lactation differs substantially from the later lactation periods; the intermediate period of the lactation is the most representative of the whole lactation, with the highest correlations with any other time period; and the correlation between the beginning and the end of the lactation can be rather low, nonetheless still positive. Therefore, we chose to account for the two gradients in the random regression model, on DIM and on THI. This choice corresponds to the model which fits best our objective to evaluate the genetic-by-environment interactions, although this model is computationally very demanding and limits the size of the analyzed dataset. Nevertheless, this model was relevant, since the estimates of heritability varied considerably with DIM (0.06 to 0.33 for yields, 0.08 to 0.56 for contents and 0.03 to 0.16 for SCS). In spite of these large variations in heritability, it is important to note that the estimates were mostly within the range of expected values over a large part of the lactation.

Although the present random regression model takes the individual variations in the shape of the lactation curve into account, these variations are only briefly addressed in the “Results” and “Discussion” sections of this paper, because our study focused on individual variability related to THI. For the same reason and due to computational limitations, only two Legendre polynomials were considered for DIM, which is enough for the present purpose but probably too limited for a complete fit of the individual trajectories across DIM. However, the trajectories of the variances and heritabilities along the time gradient have been verified and are consistent with the values found in the literature, with heritability estimates increasing with DIM [28, 40]. As in Brügemann et al. [40], the effect of DIM on heritabilities was more pronounced than the effect of THI, and genetic correlations between distant DIM can reach values lower than 0.50.

In agreement with the results of Brügemann et al. [40] on PY, Bohlouli et al. [19] on MY, and Carabaño et al. [28] on milk, fat and protein yields, our estimates of heritabilities tended to decrease as the temperatures increased, mostly due to the increase in residual variance. This increase in residual variance by itself is frequently considered as an indicator of stress. However, the results available in the literature on the variation in heritabilities for production traits with THI are divergent. Contrary to our trends, some authors highlighted an increase in heritability with increased THI [35, 41]. It is important to note that these increases were observed for high THI, i.e. above 72 (78 for FY), which are very rare THI values in our dataset. Moreover, Aguilar et al. [41] and Ravagnolo and Misztal [35] used the broken line model with a THI threshold fixed at 72 for all cows, which does not allow to estimate variations in heritability below this threshold. Since our dataset contained few performances recorded for THI values higher than 70, the decrease in heritability observed in our study needs to be confirmed.

Among the results for SCS, Bohlouli et al. [19], Hammami et al. [42] and Santana et al. [22] reported heritability estimates for German, Belgian and multiparous Brazilian Holstein cows, respectively, which increased as THI increased, in agreement with our results. This increase in heritabilities may be explained by the increase in SCS with THI which may favor the expression of genetic variability. In Spanish Holstein, in addition to the increase in heritability for SCS at higher temperatures, Carabaño et al. [28] found that it also increased at the lowest temperatures. Similarly, in Iranian Holstein, Atrian-Afiani et al. [43] observed the highest estimates of heritability in the coolest production zone that they studied. Such results at the lowest temperatures were not observed in our study.

In agreement with our results, Aguilar et al. [41] for MY, Cheruiyot et al. [44] for milk, fat and protein yields, and Brügemann et al. [40] for PY, found permanent environmental variances larger than the genetic variances and a declining trend of these permanent environmental variances with THI, in line with the increase in residual variances.

Some trajectories of variances and heritabilities showed a rebound at the highest THI values. It can be hypothesized that these upward bounces are due to a difference in the genetic determinism of the traits at the highest THI, and to a higher genetic variability at THI above 70 as in Aguilar et al. [41], Cheruiyot et al. [44] and Ravagnolo and Misztal [35]. However, we believe that a more likely hypothesis is that these upward bounces are due to the scarcity of the data recorded at extreme THI (only 3% of the performances were recorded at THI ≥ 70), leading to a possible misfit of the model at high THI. As mentioned before, these border effects are well-known drawbacks of the use of polynomials in regression models.

Within DIM, our estimates of genetic correlations between THI conditions are very high and in line with Brügemann et al. [40] and Cheruiyot et al. [44]. Considering correlations between a neutral zone (10 °C) and hot temperatures (30 °C), Carabaño et al. [28] estimated genetic correlations ranging from 0.85 to 0.96 for milk, fat and protein yields and SCS also in agreement with our results. However, due to a higher prevalence of warm temperature conditions encountered in their dataset, these authors highlighted that genetic-by-THI interactions can be much stronger (with estimates of genetic correlations ranging from 0.24 to 0.86 between extreme temperatures, − 1 vs. 34 °C, depending on the model), especially for fat and protein contents and SCS. In many studies, MY was not the best trait to identify variability in response to heat stress with genotype [28, 42, 45]. Among all the traits studied here, PY and SCS in L2 are those with the largest genetic-by-THI interactions.

Within-trait and DIM, the permanent environmental correlations between adjacent and between distant THI were also high [minimum estimates of permanent environmental correlations at DIM = 150d were 0.89 for THI ranging from 50 to 70, for FC in L1, (see Additional file 2: Fig. S2)], which indicates that the permanent effect of the cows did not display strong interactions with THI.

In the literature, the estimates of the genetic correlations between the intercept (general level of production) and the slope of decay above the heat tolerance threshold were all negative for production traits, ranging from − 0.16 to − 0.56 on MY, PY and FY in various Holstein populations [33, 42, 44], and (see review in [37]). For SCS, Hammami et al. [42] reported a positive genetic correlation (+ 0.25) between intercept and slope. In our study, the breeding value at a THI of 50 was chosen instead of the intercept to avoid structural negative correlations due to the model. In spite of this change in definition, the results were in the same order of magnitude, which is explained by the very high correlation between the intercept and production level at a THI of 50. Our results are therefore in line with those available in the literature with a negative correlation for production traits (animals with the highest EBV are the most affected by heat stress) and a positive correlation for SCS (animals with the highest EBV, and therefore the highest somatic cell counts, are also the most affected during heat stress, with an even higher somatic cell count than others). However, these unfavorable correlations were, generally, more pronounced in our study.

Considering the slope as a heat tolerance trait, many authors conclude that there is an antagonistic correlation between production traits currently under selection and heat tolerance [26, 28, 33, 35]. However, on the one hand, these unfavorable genetic correlations between EBV at THI 50 and the slope at THI 70 for production traits require further biological interpretation. A key question is the biological interpretation of the slope as an adaptation indicator. Reducing production during heat stress can be a protection mechanism to favor other biological functions (survival, immune response, reproduction, etc.). Therefore, further investigations are required to estimate the evolution of the genetic correlation between production and functional traits along the THI gradient and to confirm or refute this hypothesis. On the other hand, the positive genetic correlations between EBV at THI 50 and the slope at THI 70 for SCS means that selecting for mastitis resistance under the current conditions is relevant for future warmer conditions.

## Conclusions

Heat stress induces a decline in the performance of all traits (decrease in yields and contents, and increase in SCS), but they behave differently regarding the definition of neutral temperature. Although there is a systematic decline, the relative effect remains limited, i.e. close to -10%. The climatic conditions encountered in France are not, or not yet sufficiently extreme to observe a very large drop in performances. Within the range of THI observed in this study, interactions between genotype and THI remain limited, with genetic correlations higher than 0.8 and frequently above 0.9 across conditions. These results suggest that the ranking of animals is not severely affected in warmer conditions, but they can also be interpreted as a limited flexibility of the animals to adapt to even more severe conditions. Nevertheless, a number of outlier bulls show strong slopes for THI sensitivity and, thus, it would be interesting to examine their high sensitivity or, even more relevant, their capacity to adapt and cope with heat.

## Supplementary Information

**Additional file 1: Figure S1. **Evolution of additive genetic variances for PY in first (L1) and second (L2) lactation, between 30 and 300 days-in-milk.

**Additional file 2: Figure S2.** Estimates of permanent environmental correlations within trait at different THI for milk yield (MY), somatic cell score (SCS), fat and protein contents (FC and PC) and fat and protein yields (FY and PY) in first (L1) and second lactation (L2). Results are given at 150 days-in-milk.

## Data Availability

The phenotypic and pedigree data originated from the French National Animal Breeding database which is used for selection purposes. The data are owned by the French farmers and restrictions apply to their availability. The Safran meteorological database can be obtained from Météo-France for research purposes.
