# Supplementary material for: Estimation of genotype by temperature-humidity index interactions on milk production and udder health traits in Montbeliarde cows
Source: Genet Sel Evol. 2023 Jan 19;55:4. doi: 10.1186/s12711-023-00779-1 (PMC9854084; doi:10.1186/s12711-023-00779-1)
Supplement: Supplementary file 1 — Additional file 1: Figure S1. Evolution of additive genetic variances for PY in first (L1) and second (L2) lactation, between 30 and 300 days-in-milk. [file 12711_2023_779_MOESM1_ESM.pdf]

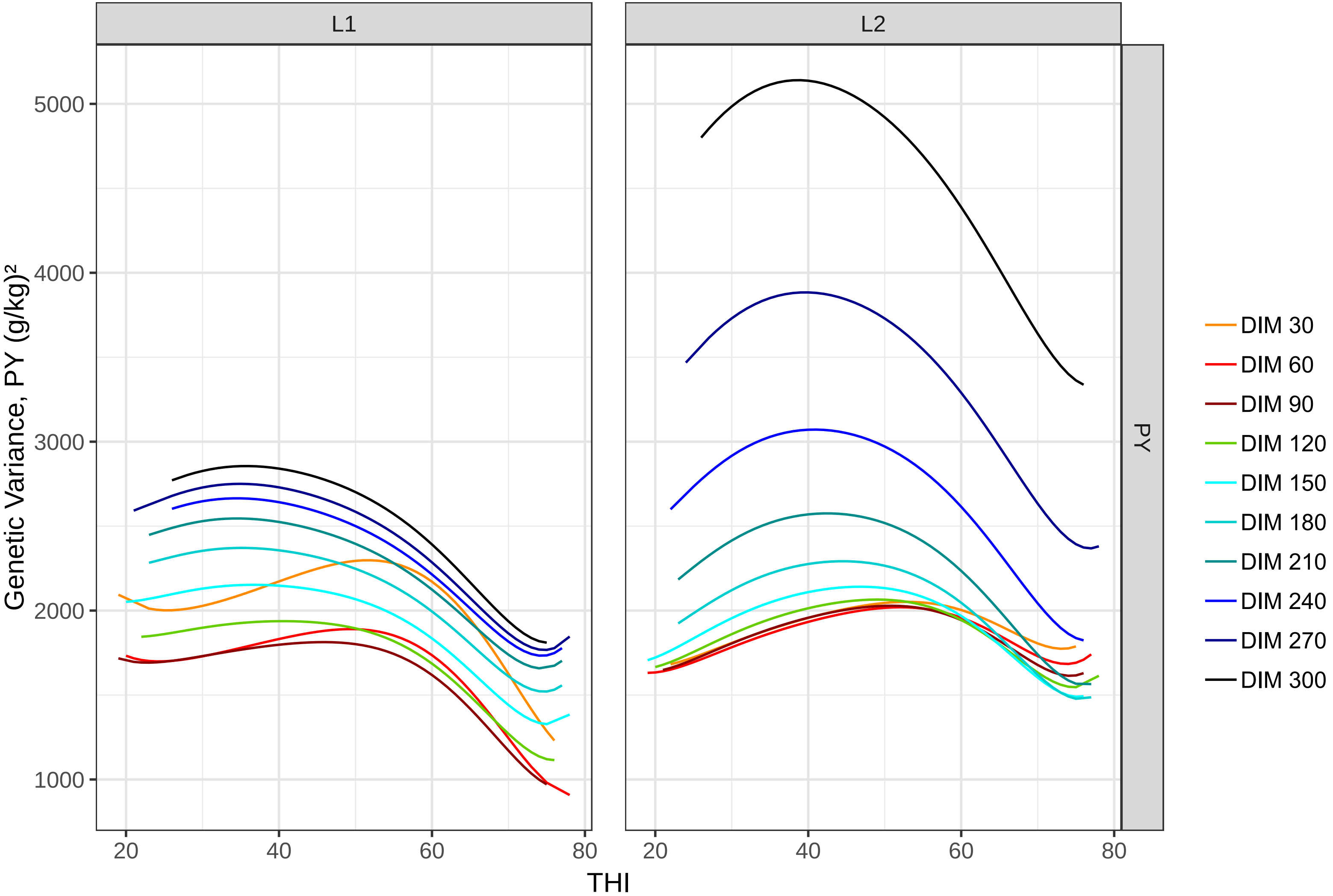

Additional file 1: Figure S1 Evolution of additive genetic variances for PY in first (L1) and second (L2) lactation, between 30 and 300 days-in-milk.
