# Supplementary material for: Estimation of genotype by temperature-humidity index interactions on milk production and udder health traits in Montbeliarde cows
Source: Genet Sel Evol. 2023 Jan 19;55:4. doi: 10.1186/s12711-023-00779-1 (PMC9854084; doi:10.1186/s12711-023-00779-1)
Supplement: Supplementary file 2 — Additional file 2: Figure S2. Estimates of permanent environmental correlations within trait at different THI for milk yield (MY), somatic cell score (SCS), fat and protein contents (FC and PC) and fat and protein yields (FY and PY) in first (L1) and second lactation (L2). Results are given at 150 days-in-milk. [file 12711_2023_779_MOESM2_ESM.pdf]

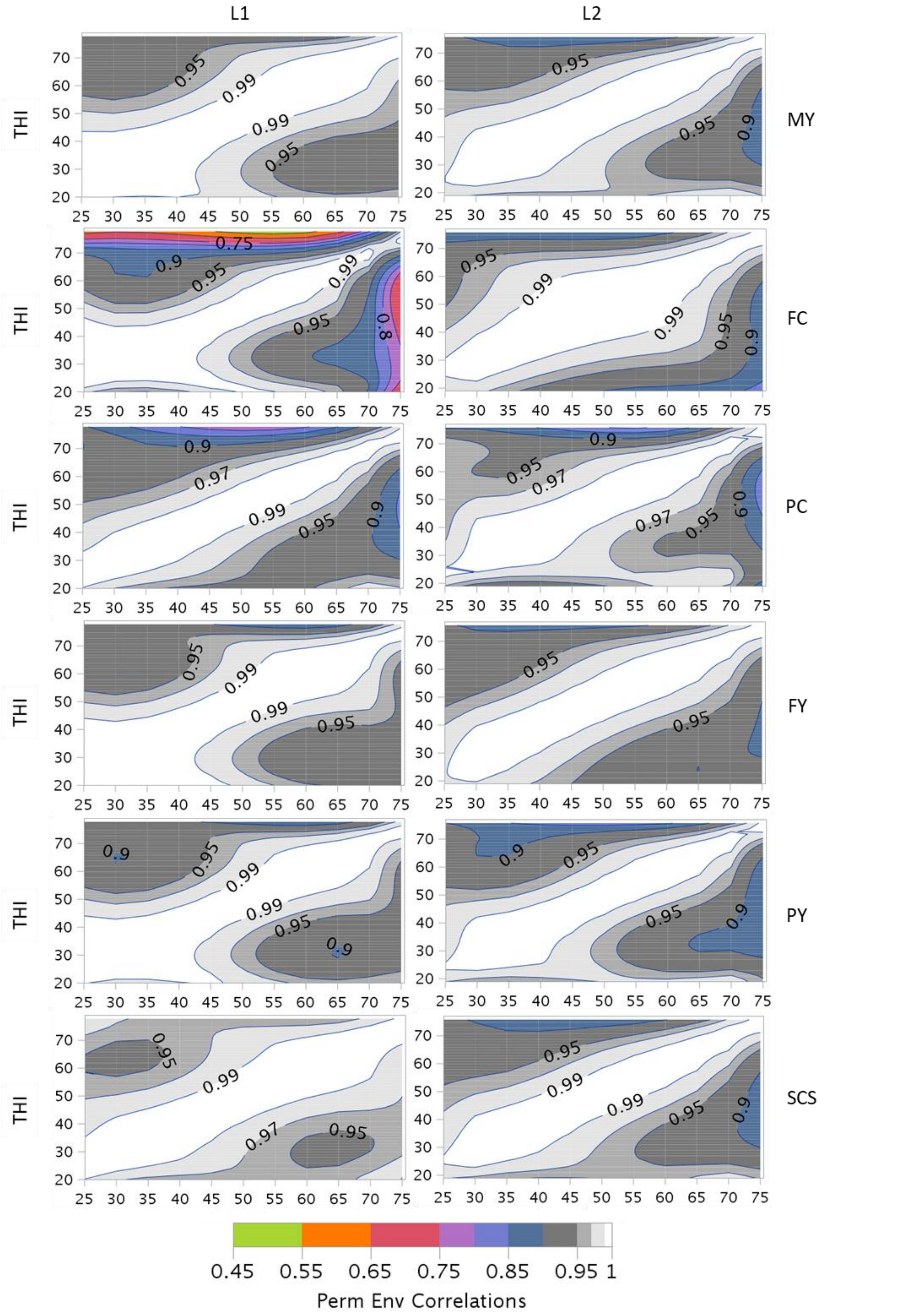


Additional file 2: Figure S2 Estimates of permanent environmental correlations within trait at different Temperature-Humidity Index (THI) for milk yield (MY), somatic cell score (SCS), fat and protein contents (FC and PC) and fat and protein yields (FY and PY) in first (L1) and second lactation (L2). Results are given at 150 days-in-milk.
